# Consumer traits of common beans: a global and regional perspective on seed coat darkening, cooking time, protein, and mineral content

**DOI:** 10.3389/fnut.2025.1658338

**Published:** 2025-09-26

**Authors:** Julius Mbiu, Teshale Assefa, Clare Mukankusi, Jean Claude Rubyogo, Mashamba Philipo

**Affiliations:** ^1^School of Life Sciences and Bio-engineering, The Nelson Mandela African Institution of Science and Technology (NM-AIST), Arusha, Tanzania; ^2^Selian Agricultural Research Centre, Tanzania Agricultural Research Institute (TARI), Arusha, Tanzania; ^3^Alliance of Bioversity International and International Center for Tropical Agriculture (CIAT), Arusha, Tanzania; ^4^Alliance of Bioversity International and International Centre for Tropical Agriculture (CIAT), Kampala, Uganda; ^5^Alliance of Bioversity International and International Center for Tropical Agriculture (CIAT), Nairobi, Kenya

**Keywords:** proanthocyanidins, seed coat darkening, cooking time, protein content, mineral content, common Bean, consumers’ traits

## Abstract

Common beans (*Phaseolus vulgaris* L.) are a cornerstone of global nutrition, offering a sustainable source of protein, micronutrients, and bioactive compounds. This review synthesizes current research on critical consumer traits—seed coat darkening, cooking time, protein, and mineral content—highlighting their genetic, biochemical, and environmental determinants. Seed coat darkening, driven by proanthocyanidin oxidation and regulated by genes like *J*, *sd*, and *Psd*, significantly impacts marketability, while cooking time variations (19–271 min across genotypes) influence regional preferences and nutritional outcomes. Biofortification and low-phytic acid (lpa) breeding strategies enhance mineral bioavailability, addressing deficiencies in Sub-Saharan Africa and South Asia. Regional disparities in consumer preferences, such as the demand for fast-cooking yellow beans in East Africa, underscore the need for tailored breeding programs. Climate change poses challenges to yield and nutrient retention, necessitating climate-resilient varieties. This review proposes integrating genomics, marker-assisted selection, and postharvest innovations, for developing beans that align with consumer needs, cultural practices, and sustainability goals. This is the first synthesis linking seed coat biochemistry to regional preferences.

## 1 Introduction

Common beans (*Phaseolus vulgaris* L.), also known as string beans, french beans, haricot beans, and snap beans, are herbaceous annual plants cultivated worldwide for multiple uses. These include dry beans, shell beans (harvested at physiological maturity), and green pods. In some regions, their leaves are consumed as vegetables, and the straw is used as animal fodder (1).

Common beans display remarkable genetic and morphological diversity. They are grouped into two major gene pools, Andean and Mesoamerican each encompassing a wide range of commercial seed types with varying sizes, shapes, and color types (123). Their adaptability to diverse environments, combined with high nutritional value, affordability, and long shelf life, has established dry beans as the most widely consumed legume globally (2).

The crop is primarily cultivated for their immature pods, green shelled seeds, and mature dry seeds. Common beans are a vital source of nutrition in many parts of the world, especially in Latin America and Africa (124, 133). They are rich in high proteins (20–28%), iron (70 mg/kg), zinc (33 mg/kg), and vitamin A, offering a nearly complete nutritional profile alongside antioxidant benefits (1).

In addition to their nutritional contributions, common beans play a significant role in food security and sustainable agriculture. Their nutrient density and postharvest stability make them an essential crop, particularly in developing countries where they are central to traditional farming systems, such as those in sub-Saharan Africa (3, 128).

Common beans also carry cultural importance, shaping food traditions and culinary practices across the globe. Their consumption continues to rise in most regions, with the exception of Central and East Asia (4). A wide variety of cultivars navy, pinto, carioca, great northern, yellow, black, red, pink, and kidney beans their diversity in seed color, pattern, size, and shapes (5).

From a consumer perspective, preferences for common beans are influenced by characteristics such as seed color, size, cooking time, and gravy quality (6). Nutritional and sensory traits iron content, seed coat darkening, and digestibility are closely linked and significantly affect consumer acceptance and market demand (7, 8).

Studies have shown that cooking time is a key factor in consumer choices, as highlighted in Malawi (135) and similar preferences are observed in Tanzania, where local markets offer a wide variety of beans differing in appearance, cooking quality and ease of digestion (6, 127).

Despite the global significance of common beans and the availability of numerous improved varieties, there remains a limited understanding of how specific consumer traits—particularly seed coat darkening, cooking time, protein content, and mineral content—affect consumer preferences across different regions. Most existing studies tend to focus on agronomic performance or yield-related characteristics, often neglecting the postharvest and consumption-related traits that directly influence marketability and acceptability. Moreover, the relationship between consumer traits and nutritional quality is not consistently integrated into breeding priorities, especially in sub-Saharan Africa. There is also insufficient data comparing consumer preferences across global and regional contexts, which limits the ability to develop universally or locally acceptable varieties.

Extensive research exists on consumer preferences for common beans, including economic studies on the influencing factors (6) and localized sensory research on attributes like cooking time and color (9). However, a crucial gap remains in consolidating evidence on operational strategies for scaling up preferred bean varieties to create a systemic impact across African food systems.

This review seeks to deliver an in-depth analysis of essential consumer traits in common beans, by examining these key traits from both global and regional perspectives, the review will investigate the underlying mechanisms, variability, and implications for consumer acceptance. Additionally, it will identify challenges associated with these traits and propose future research directions aimed at enhancing them through breeding, biotechnology, and sustainable practices. The analysis acknowledges that these traits differ across regions due to variations in cultural preferences, dietary requirements, and environmental conditions.

## 2 Literature search methodology

This review compiled global studies on consumer traits of common beans (*Phaseolus vulgaris* L.) by systematically searching peer-reviewed articles from 2000 to 2024. The databases used were Web of Science, Scopus, PubMed, and Google Scholar, with keywords such as proanthocyanidins, seed coat darkening, postharvest darkening, cooking time, protein content, mineral content, biofortification, common bean genetics, consumer preferences, and regional adaptation. The inclusion criteria focused on studies examining genetic, biochemical, and environmental factors affecting seed coat darkening, cooking time, protein content, and mineral content (Fe, Zn); data from various agroecological regions (Sub-Saharan Africa, Latin America, South Asia); and articles written in English. Exclusion criteria ruled out non-empirical reports, studies on non-Phaseolus legumes, and duplicate datasets. Additional sources were identified through snowballing from reference lists. Data extraction emphasized genetic mechanisms (e.g., *J, sd, Psd* genes; QTLs like *CT-Pv03*), socioeconomic factors affecting consumer acceptance, and implications for climate resilience.

## 3 Results and discussion

### 3.1 Seed coat darkening (postharvest darkening)

Seed coat darkening, or postharvest darkening (PHD), is a common visual defect in *Phaseolus vulgaris* where the seed coat gradually darkens from a lighter to a darker hue during storage time (10). This natural process primarily results from the oxidation of phenolic compounds, especially anthocyanins. Seed coat color significantly impacts consumer acceptance; lighter-colored beans are generally preferred, making PHD a critical factor in marketability (11). Susceptibility to PHD reduces the commercial value of dry beans, leading to economic losses for producers, exporters, and vendors (12).

Based on the rate and extent of PHD, beans are categorized into three phenotypes: Regular Darkening (RD), exhibiting rapid darkening; Slow Darkening (SD), darkening at a reduced rate; and Non-Darkening (ND), showing minimal to no darkening (2). These classifications are particularly relevant in market classes such as pinto beans.

Genetic and environmental factors influence the rate of darkening (13). The *J* gene determines whether darkening occurs, with the recessive *jj* genotype resulting in ND beans. The *sd* gene influences the rate of darkening; the recessive *sdsd* genotype results in SD, while the dominant *Sd* allele leads to faster darkening. Consequently, a key objective in bean breeding programs is developing SD cultivars, which maintain their lighter color longer, enhancing marketability and economic value (14). Seed coat color, along with hilum ring and corona characteristics, are crucial for market acceptance; therefore, susceptibility to PHD is a critical consideration in bean breeding and selection (130, 131).

#### 3.1.1 Mechanisms and causes

Seed coat darkening involves complex biochemical and genetic mechanisms. The primary biochemical process is the oxidation of proanthocyanidins (PAs), also known as condensed tannins, into reactive quinones, leading to seed coat browning (15).

**Figure 1:**
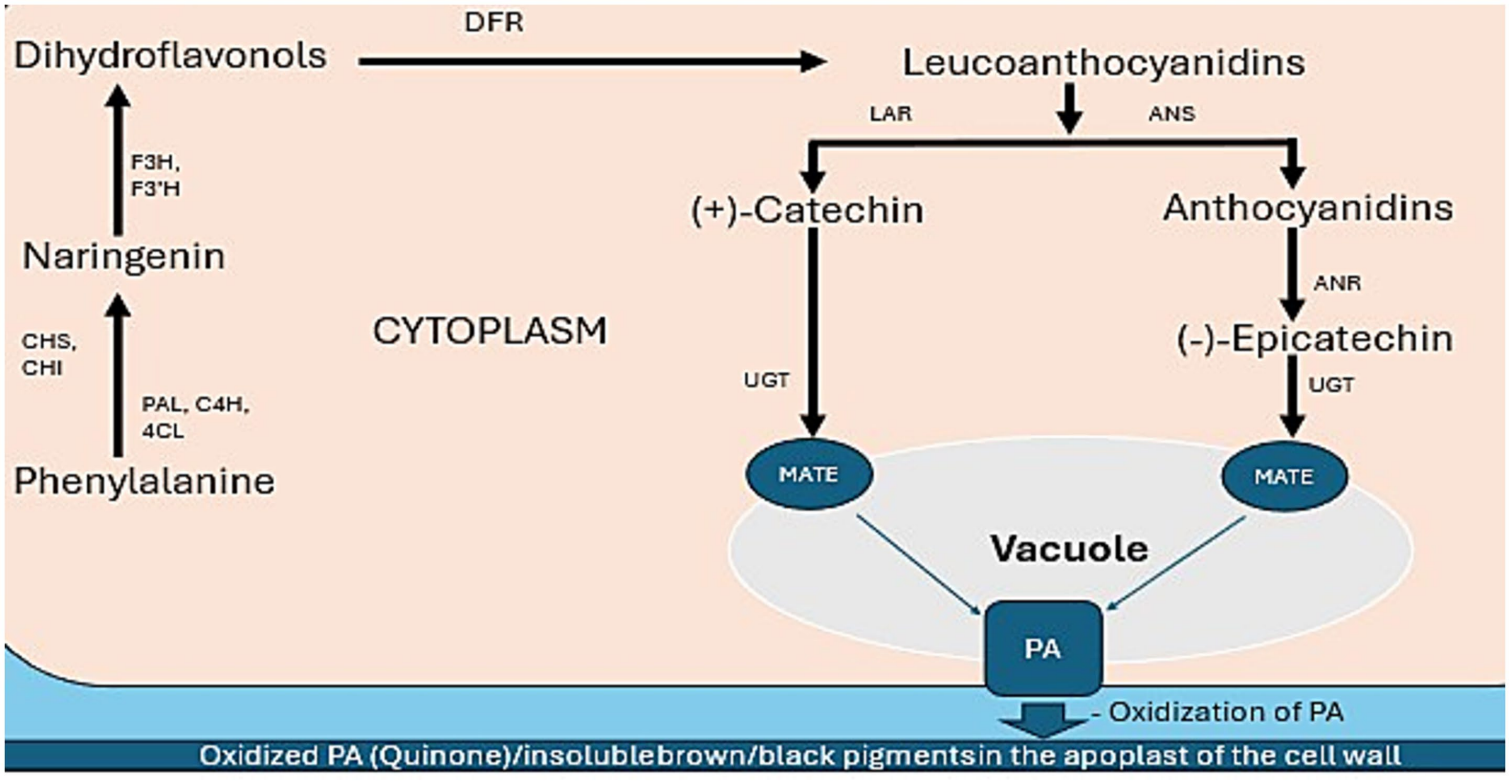
Proposed proanthocyanidin biosynthetic pathway in bean seed coats. Key enzymes: PAL (Phenylalanine ammonia lyase), C4H (Cinnamate-4-hydrolase), 4CL (4-coumarate-CoA ligase), CHS (Chalcone synthase), CHI (Chalcone isomerase), F3H (Flavanone 3-hydroxylase), F3’H (Flavonoid 3′-hydroxylase), DFR (Dihydroflavonol reductase), LAR (Leucoanthocyanidin reductase), ANR (Anthocyanidin reductase), ANS (Anthocyanidin synthase), PA (Proanthocyanidin), MATE(Multidrug and toxic compound extrusion protein), The ovals indicate membrane transporters.

The biosynthesis of proanthocyanidins (condensed tannins) in common bean (*Phaseolus vulgaris*) seed coats involves five key stages (Figure 1), culminating in seed coat darkening (16, 17).

1. Phenylpropanoid pathway: The pathway initiates with the amino acid phenylalanine, convert to naringenin and key enzymes involved in are phenylalanine ammonia lyase (PAL), cinnamate-4-hydroxylase (C4H), and 4-coumarate-CoA ligase (4CL) catalyze early conversions (134). chalcone synthase (CHS) and chalcone isomerase (CHI) form the core flavonoid precursor naringenin (136).
2. Flavonoid pathway (dihydroflavonol formation): Naringenin is hydroxylated by flavanone 3-hydroxylase (F3H), flavonoid 3′-hydroxylase (F3’H), and flavonoid 3′,5′-hydroxylase (F3’5’H) to produce dihydroflavonols. DFR (dihydroflavonol reductase) reduces dihydroflavonols to form leucoanthocyanidins (126, 136).
3. Monomer synthesis. In this stage the Leucoanthocyanidins diverge into two pathways: the first path leucoanthocyanidin reductase (LAR) enzyme is acted to produces (+)-catechin (125), while the second path anthocyanidin synthase (ANS) converts leucoanthocyanidins to anthocyanidins (e.g., cyanidin). Anthocyanidin reductase (ANR) then reduces cyanidin to form (−)-epicatechin, the dominant PA monomer in colored beans (125).
4. Polymerization and transport: Monomers are glycosylated by UDP-glucosyltransferases (UGTs) and transported into the vacuole with multidrug and toxic compound extrusion (MATE) transporters (137). Within the vacuole, monomers undergo polymerization to form proanthocyanidin (PA) polymers (16, 137).
5. Oxidation and seed coat darkening: PAs are located in the apoplast (cell wall/extracellular space) in mature seeds (17). Polyphenol oxidases (PPOs) oxidize the PAs in this compartment (17) leads to quinone formation and subsequent polymerization into insoluble brown/black pigments known as melanins, causing seed coat darkening during storage or aging (17).

This process depends on PA availability and the activity of polyphenol oxidases (PPOs) like laccase and tyrosinase. While not a PPO, peroxidase may also contribute to oxidation processes and play a role in secondary metabolism and cell death regulation (18). PPO plays a crucial role in pigment synthesis and degradation. These enzymes catalyze the oxidation of phenolic compounds into quinones, which polymerize into brown pigments (19). While PPO activity is generally associated with undesirable browning, it may also play a role in secondary metabolism and cell death regulation.

It is important to note that while anthocyanins are not the direct primary substrates for PPOs, they can be degraded through co-oxidation mechanisms during PPO-catalyzed reactions, contributing to color loss and browning (20).

The genetics of seed coat darkening involve at least two unlinked major genes. The *J* gene determines the potential for darkening, with the recessive *jj* genotype resulting in non-darkening seeds. The *sd* gene influences the rate of darkening, with the recessive *sdsd* genotype darkening more slowly than individuals with the dominant *Sd* allele (21). Additionally, the *Psd* gene, an allele of the *P* (Pigment) gene, is responsible for the slow-darkening trait in pinto beans (10). Understanding these genetic mechanisms has facilitated the development of slow-darkening (SD) bean varieties, which not only retain their visual appeal longer but also offer practical advantages. SD beans cook 30% faster and provide 2–7 times more bioavailable iron than regular-darkening pinto beans (8). This research has important implications for breeding programs, potentially enhancing both the quality and nutritional value of pinto beans product (8, 22).

Understanding the biochemical processes that change seed coat color enables breeders to develop cultivars with stable seed coat color (23). Seed coat darkening is more pronounced in market classes like yellow, pinto, carioca, and cranberry beans and is linked to genetics, growing environment, postharvest storage, and chemical composition (5). Red and black beans within the Middle American gene pool have higher polyphenols in the seed coat extract than cream, yellow, or pink beans (12). Phenolic compounds, including phenolic acids, flavonoids, PAs (condensed tannins), and coumarins, significantly affect bean seed color (5, 20).

**Figure 2:**
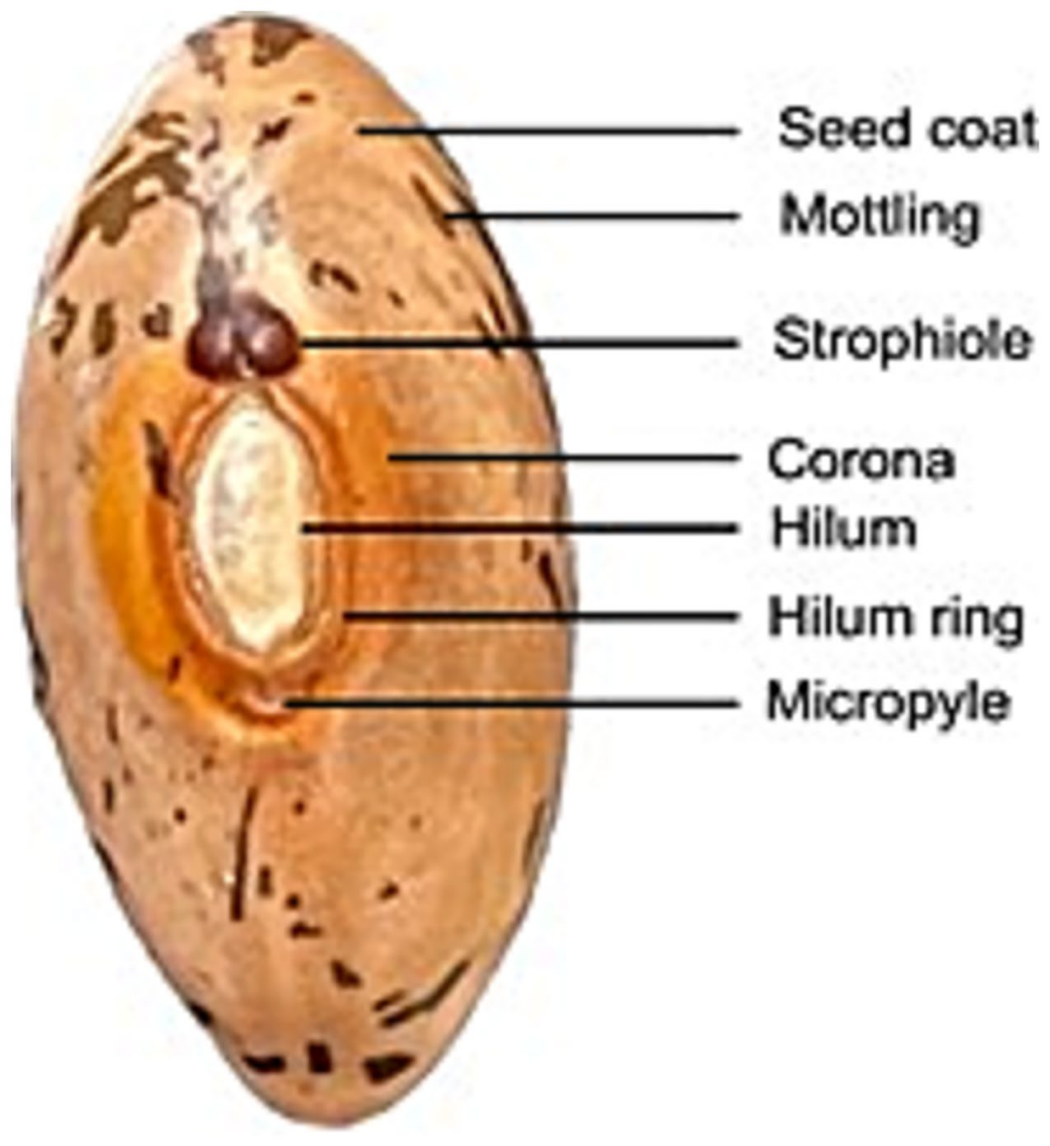
Area specific PA-level variation in a seed of pinto bean cultivar CDC Pintium. Reproduced from Islam and Dhaubhadel (2), licensed under CC BY 4.0.

PAs accumulate in seed coat tissues, playing a role in seed germination, viability, and protection against biotic and abiotic stresses, ensuring long-term storage potential (2). In pinto beans, PAs are visible in distinct locations on the seed coat, including the base, mottling, hilum ring, corona, micropyle, and strophiole (Figure 2). Variations in brown shades indicate different PA deposition and oxidation levels. Seed coat background and hilum color affect canning and storage quality (2).

The phenolic composition is specific to each bean genotype. For example, black beans are notably high in anthocyanins such as petunidin-3-glucoside and malvidin-3-glucoside, which give them their dark color. In contrast, pinto and cranberry beans exhibit unique patterns of proanthocyanidin polymers (16, 20). Additionally, yellow bean varieties, which are highly valued in East Africa, owe their vibrant color to carotenoids like lutein and zeaxanthin, rather than phenolic compounds (5).

Environmental factors, particularly temperature during seed maturation, significantly influence PPO activity and seed coat darkening. For example, low temperatures during seed maturation in *Arabidopsis* increase phenylpropanoid gene expression and procyanidin concentrations (24). Similarly, in common beans, genotype × environment (G × E) effects are significant for seed color, with beans grown in Michigan being darker than those from Nebraska (25). RD cultivars of pinto beans accumulate higher PA levels than SD cultivars (26). The *Sd* gene, identified as *Psd*, encodes a bHLH transcription factor involved in PA biosynthesis. Recent work by Parker et al. (129) reveals that MYB-bHLH-WD40 complexes regulate seed color patterns in common beans, with temperature modulating anthocyanin biosynthesis. This underscores the need for breeding programs to account for genotype × environment × management (G × E × M) interactions in maintaining color stability.

Post-harvest storage conditions, including temperature, light, humidity, and storage time, can contribute to PHD by altering the physical and chemical characteristics of the seed coat. Storage temperature and duration affect color and phenolic content (27) and cooking quality (28) of faba bean seed (5). Humidity and drying time after harvest also play a role (13). Higher temperatures (20 or 30 °C) and longer storage (120 or 180 days) generally result in darker seeds with increasing redness compared to seeds stored at 6 °C or for 60 days (5).

High temperature, moisture, and radiation in the tropics accelerate postharvest disorders, affecting integument color and bean hardness (29). During storage, superoxide dismutase (SOD) activity and lipid peroxidation (LP) increase in the cotyledon, more significantly in rapid-darkening genotypes (29).

**Figure 3:**
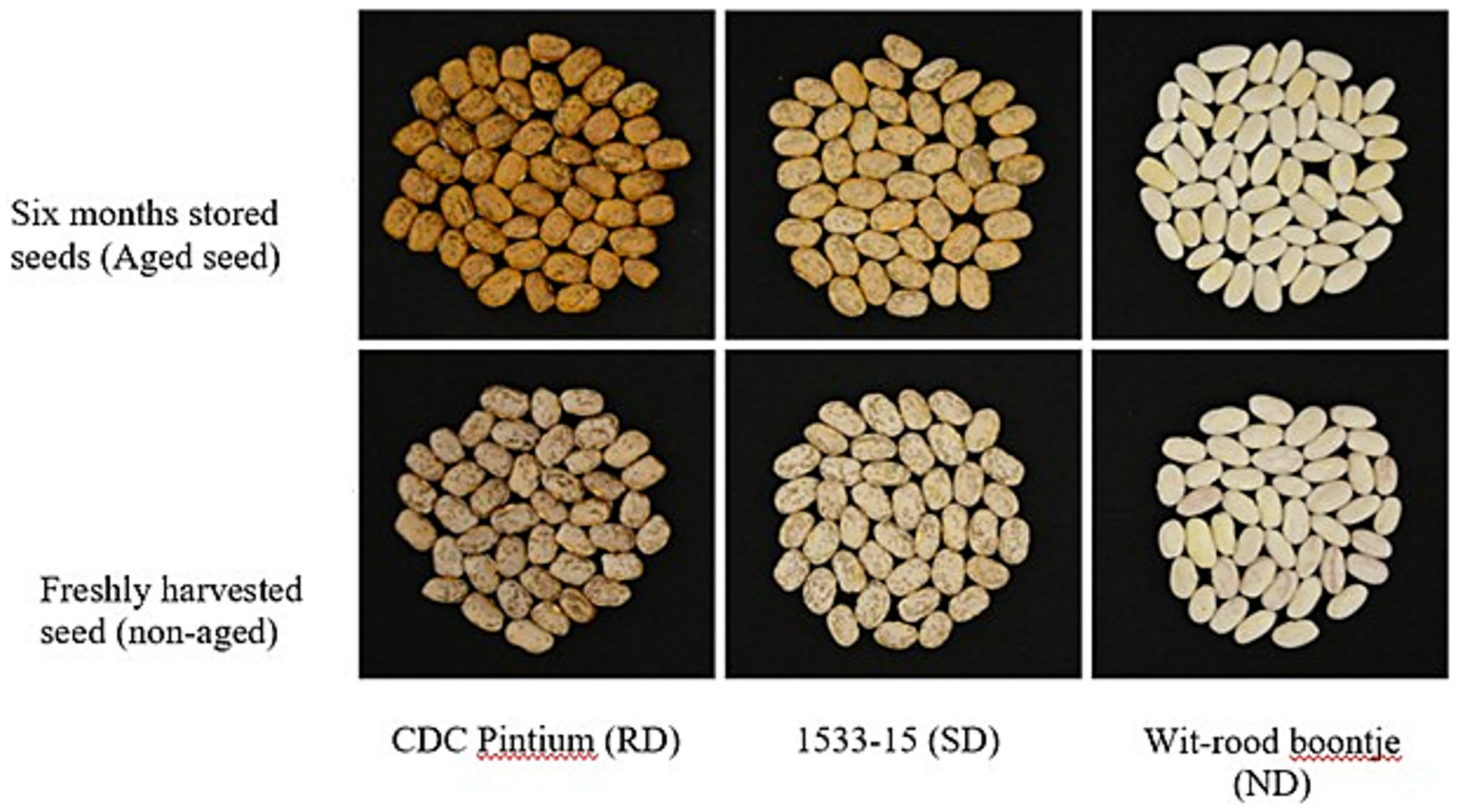
Postharvest seed coat darkening in pinto beans. Picture of three different pinto bean varieties: CDC Pintium (RD), 1533-15 (SD) and cranberry like Witrood boontje (ND), freshly harvested seeds (non-aged) and seeds stored for 6 months at room temperature. Reproduced from Islam and Dhaubhadel (10), licensed under CC BY 4.0.

The rate and extent of darkening depend on cultivar., storage conditions, and processing methods. For example, the RD pinto bean cultivar CDC Pintium (Figure 3) darkens faster than the SD cultivar 1533-15 (12). Higher temperatures, humidity, and light accelerate darkening (2, 23). Canning or freezing may slow down or minimize darkening compared to regular storage (12).

#### 3.1.2 Effects and impact of regular darkening in common beans

Common beans exhibit considerable diversity in color, size, and shape, catering to a wide range of consumer preferences (11, 30). The color of the seed coat, hilum ring, and corona is critical for marketing, as these traits closely align with consumer choices (132), Xiong et al. (5). Moreover, susceptibility to postharvest darkening (PHD) diminishes seed value, further influencing consumer preferences (25). Preferences for specific bean types vary regionally (13).

Darkened beans are often associated with prolonged cooking times altered water absorption, affecting texture and palatability and perceived lower nutritional quality, particularly in regions with limited fuel availability (2, 10, 11, 21, 31, 32). Due to high levels of proanthocyanidins which can hinder nutrient absorption, particularly iron, potentially raising health concerns (2, 10), resulting in product downgrading and reduced market value (2, 13, 23, 31). This perception diminishes consumer desirability product (8, 22).

#### 3.1.3 Breeding strategies to address seed coat darkening in common beans

The inheritance of post-harvest darkening (PHD) involves at least two unlinked major genes: the J gene, which prevents darkening entirely with the recessive *jj* genotype, and the *sd* gene, which slows the darkening process (21). Understanding these genetic mechanisms has facilitated the development of SD bean varieties that not only maintain their visual appeal longer but also provide additional benefits, such as faster cooking times and improved iron bioavailability (8).

The development of gene-specific markers and accelerated darkening protocols, such as the UVC light method, can assist breeders in selecting for the SD trait more efficiently (10, 33). These advancements equip breeders with effective tools to create improved bean varieties and retain market value during extended storage periods. However, further studies are necessary to fully understand the relationships between growing conditions, seed color, and polyphenolic profiles (25).

Traditional breeding has effectively improved various traits in common beans, such as disease resistance and drought tolerance (34). However, for complex traits like seed coat darkening, which involve multiple genes and environmental interactions, traditional methods may face limitations in efficiency and precision.

Marker-assisted selection (MAS) has emerged as a powerful tool for enhancing various traits in common beans. This approach enables breeders to select for desired traits using closely linked molecular markers, even in the absence of trait expression (35). MAS has been particularly successful in stacking disease resistance genes and QTLs (36, 37). For seed coat darkening, MAS could be utilized if markers linked to the controlling genes are identified.

Recent advances in genomic technologies have paved the way for more sophisticated breeding approaches. Genomic selection (GS) has emerged as a valuable method for improving complex traits controlled by many genes with small effects in plant breeding programs (38). Studies have shown that GS can achieve higher prediction accuracies compared to traditional marker-assisted selection for quantitative traits (39). This method, combined with high-throughput genotyping platforms, could accelerate genetic gains and facilitate the development of varieties with desired seed coat characteristics (40).

To facilitate the development of SD varieties, a quick, consistent, economical and most effective method of accelerate darkening for screening purposes has being established which uses ultraviolet C (UVC) light, and does not negatively impact seed germination (33).

While traditional breeding methods have laid the foundation for common bean improvement, integrating molecular approaches like MAS and genomic selection offers promising avenues for addressing complex traits such as seed coat darkening. These advanced strategies can enhance breeding efficiency and precision, leading to the rapid development of varieties with improved seed coat qualities.

Breeding programs have developed SD bean varieties characterized by a recessive gene (Psd), which modifies procyanidin production to delay darkening (8, 10). These SD varieties not only cook 30% faster but also provide 2–7 times more bioavailable iron compared to regular-darkening (RD) varieties (8).

Altering genes like Psd may inadvertently affect stress tolerance, as proanthocyanidins protect against biotic and abiotic threats (2). Breeding programs must monitor off-target impacts, such as linkage drag reducing disease resistance (e.g., impacting resistance to angular leaf spot) (41).

To avoid these issues, the Optimal Contribution Selection (OCS) strategy optimizes genetic contributions from parents to maintain genetic diversity while selecting for desired traits (42). By integrating genomic data, OCS can minimize unintended effects of seed coat color modification by selecting combinations of alleles that balance seed color with critical traits like yield, disease resistance, and nutritional quality. For example, OCS can prioritize individuals with favorable Psd alleles for slow-darkening while retaining QTLs like Fe. Zn-b06 for mineral content or CT-Pv03 for reduced cooking time (43, 44). This approach leverages high-density single nucleotide polymorphism (SNP) markers to ensure that selection for seed coat color does not compromise other agronomic traits.

### 3.2 Cooking time

#### 3.2.1 Regional cooking practices

Cooking time is a critical attribute that can significantly impact consumer preferences. Many low-income consumers favor fast-cooking beans to conserve fuel (45). Globally, common beans serve as a vital source of protein and micronutrients, particularly in Latin America, the Caribbean, and Sub-Saharan Africa (46). However, the lengthy cooking times often required can deter consumers, especially in areas dependent on costly fuelwood for cooking (46).

Different cooking methods also affect the nutritional value of beans. For instance, pressure cooking combined with soaking can enhance iron retention in the broth while diminishing it in the beans themselves (47). In Mexico, popular methods for preparing mashed beans include pressure cooking followed by mashing or frying after mashing. These techniques influence the bioaccessibility of phenolic compounds, with cooked samples showing about 50% bioaccessibility compared to only 30% in fried samples (48).

Regional cooking practices significantly shape the nutritional quality and acceptability of common beans. To maximize health benefits, it is advisable to consume both the beans and the broth (47). Additionally, developing fast-cooking varieties, such as the Manteca yellow bean, could help mitigate long cooking times and enhance iron bioavailability (46).

#### 3.2.2 Factors influencing cooking time

The cooking time of common beans is affected by a range of factors. Storage conditions, for example, play a crucial role; beans stored at high temperatures (35 °C) and humidity (80% RH) for extended periods exhibit significantly longer cooking times compared to freshly harvested beans (49). Long-term storage (up to 5 years) in tropical conditions (30–40 °C, >75% RH) can increase cooking time up to 12 times that of fresh beans (50).

Genetic factors are also pivotal in determining cooking time. Research on a recombinant inbred line population identified ten quantitative trait loci (QTLs) associated with cooking time, with three robust QTLs reducing cooking time by 11–26 min (51). A study of 206 *Phaseolus vulgaris* accessions revealed a fivefold variation in cooking time, with some beans cooking nearly 20 min faster than the average (44).

Contradictory findings exist regarding the relationship between certain bean components and cooking time. For instance, while one study found no significant link between tannins and cooking time (52), another indicated that beans with higher tannin levels were more resistant to cooking (50). Additionally, the ratio of phytic acid to calcium content in beans has been correlated with cooking times (53).

The cooking time of common beans results from a complex interplay of storage conditions, genetic traits, and chemical composition. Pre-treatment methods such as soaking, dehulling, and specific cooking solutions can further influence cooking time (54, 55). Understanding these factors is essential for developing strategies to reduce cooking times and enhance the culinary versatility of beans.

#### 3.2.3 Consumer preferences

**Figure 4:**
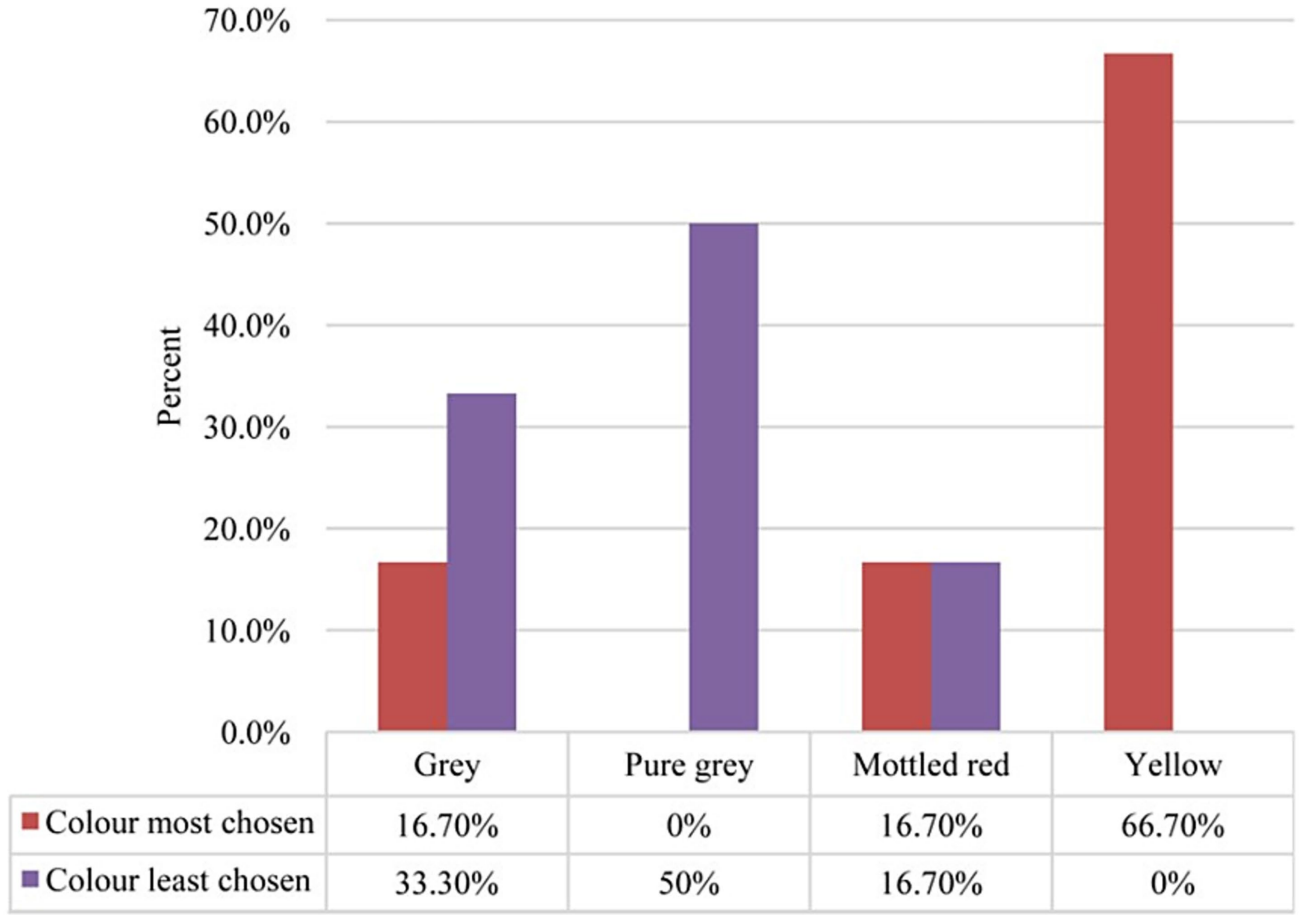
Most and least chosen common bean colour. Reproduced from Swema and Mwinuka (6), licensed under CC BY 4.0.

Cooking time is a crucial factor for consumers, particularly in developing nations where energy costs are a significant concern (1). Many consumers prioritize shorter cooking times for beans, as they are associated with lower energy expenditures (56). A study in Dar es Salaam revealed that consumers prefer yellow beans, which are linked to faster cooking by (6). Figure 4 shows that yellow is most preferred (66.7%) this dominance suggests a strong consumer preference for yellow beans. Consumers may associate yellow seeds with freshness, nutritional value, or culinary appeal while pure gray (50% least chosen) and gray (33.3% least chosen) are the least preferred colors. Understanding these preferences helps align agricultural production with market trends while promoting biodiversity in bean cultivars.

Regional differences in cooking practices exist between urban and rural areas, influenced by traditional habits, resource availability, and socioeconomic conditions. In rural settings, longer cooking times are common as these populations typically consume more pulses due to limited access to processed foods and modern cooking technologies (57).

Urban populations have better access to processed foods and modern cooking appliances, which facilitate quicker meal preparation (57). Innovations like fast-cooking bean varieties and precooked bean flours have emerged, significantly reducing cooking time (58).

This shift toward quicker cooking times is driven by the need for convenience, limited cooking fuel availability, and changing lifestyles. However, the evolution of cooking practices will continue to depend on urbanization, access to processed foods, and income levels (57). Despite these regional variations, ongoing efforts to develop quick-cooking bean varieties that maintain nutritional quality aim to bridge the gap between urban and rural cooking practices (8, 58, 59).

Fast-cooking beans are particularly favored in regions like Latin America, the Caribbean, and Sub-Saharan Africa, where the high cost and scarcity of fuelwood make quick preparation essential (46, 58). In Uganda, for instance, the demand for fast-cooking processed foods is rising, driven by changing consumption patterns, increasing incomes, and high energy costs (60). Study conducted by Asiimwe et al. (9) indicates that cooking time is highly valued and about 72% of the urban consumers were willing to pay 41–53 UGX premium for reduction in cooking time, while rural low-income households relied on slower-cooking varieties. Cooking time significantly influences consumer choices, affecting dietary nutrition and energy efficiency (61). Consumers derive considerable benefits from beans that cook quickly, saving both water and fuel (60). This preference is evident in their willingness to pay a premium for fast-cooking varieties. However, long cooking times can deter consumers globally, despite the nutritional advantages of beans (46, 58). Fast-cooking yellow beans are often marketed as a preferred option in regions reliant on expensive fuelwood (58). While specific regional preferences are noted, the trend toward fast-cooking beans appears to be a global phenomenon, with researchers focused on developing varieties that meet this growing demand across various markets (51, 61, 62). Cooking time is an important bean quality characteristic and one that bean consumers care about because longer cooking times require higher expenditures on cooking fuel (130).

#### 3.2.4 Nutritional implications

Cooking beans can alter their physical, biochemical, and nutritional properties, with prolonged cooking potentially diminishing their nutritional quality beans (1). Consumption of common beans is linked to a reduced risk of diseases such as coronary heart disease and cancer, attributed to their rich phytochemical content, including polyphenols with anticarcinogenic and antioxidant properties (63). Additionally, common beans are high in fiber.

Fast-cooking dry beans generally retain more nutrients and provide improved bioavailability compared to slower-cooking varieties. Research indicates that fast-cooking beans have higher protein and mineral retention while maintaining similar starch and fiber densities when fully cooked (58). For instance, the fast-cooking yellow bean cultivar Cebo Cela contains 20% more protein, 10% more iron, and 10% more zinc per serving than the slow-cooking Canario variety (58). Notably, a strong negative correlation between cooking time and iron bioavailability has been observed in yellow beans, with correlation values of −0.76 for pre-soaked and −0.64 for unsoaked beans across two growing seasons (46). This suggests that faster-cooking beans not only preserve more nutrients but also enhance their bioavailability.

However, prolonged storage can adversely affect mineral bioaccessibility, as both storage and cooking times can lead to increased mineral chelation by cell wall polymers (49). There is an inverse relationship between cooking time and nutrient bioavailability in common beans, with faster-cooking varieties generally offering superior nutrient retention. This finding has important implications for breeding programs aimed at developing nutritionally enriched bean varieties, particularly in regions where cooking fuel is limited or costly (46, 58). The focus on fast-cooking bean varieties could effectively address nutritional deficiencies and practical challenges in bean consumption worldwide.

In addition to preserving nutrients, the duration of cooking plays a crucial role in determining the levels of remaining antinutritional factors and allergens. Extended cooking times are typically effective in breaking down heat-sensitive antinutrients like lectins (such as phytohaemagglutinin) and protease inhibitors, which can otherwise lead to digestive issues or hinder nutrient absorption (64, 65). However, prolonged exposure to heat can also result in the loss of heat-sensitive vitamins and phenolic compounds. Cooking must be long enough to neutralize antinutrients and allergens for safety and nutrient availability, but not so long that it reduces the beans’ nutritional and phytochemical content.

Thermal degradation also directly affects color compounds. Anthocyanins are highly susceptible to heat and can break down during prolonged cooking, leading to a loss of vibrant color and potential browning. Similarly, extended cooking can degrade carotenoids like lutein in yellow beans, diminishing their visual appeal and nutritional value (5). This highlights another advantage of fast-cooking genotypes: they reduce the thermal breakdown of beneficial phytochemicals and color pigments. Developing fast-cooking bean varieties that quickly achieve this balance offers a significant advantage.

#### 3.2.5 Innovations to reduce cooking time

Innovative pre-treatment methods such as soaking, dehulling, and breeding for fast-cooking varieties have proven effective in reducing cooking times for dry beans. Soaking is a common technique that can significantly cut cooking durations. For example, soaking African yambean for 12 h reduced cooking time by approximately 50% (66). Similarly, soaking the K131 variety of dry beans for 12 h, followed by a 48-h sprouting period, resulted in the creation of fast-cooking bean flours (59). The effectiveness of soaking can be enhanced by using high pH and monovalent salt solutions, which further decrease cooking times compared to soaking in water alone (54).

Dehulling is another valuable pre-treatment method. It not only reduces cooking time but also removes antinutrients like phytates and tannins, improving overall nutritional quality. For instance, dehulling has been shown to increase protein digestibility and mineral extractability in dry beans (59). However, this process may also lead to some mineral losses since certain minerals are concentrated in the seed coat (66).

Breeding for fast-cooking varieties has also shown great promise. The Manteca yellow bean, for example, cooks in less than 20 min when pre-soaked and under 80 min when unsoaked (46). This genetic resource can be leveraged to develop a new generation of fast-cooking dry beans.

Combining these pre-treatment methods can significantly decrease cooking times while enhancing nutritional quality. For instance, a combination of soaking, dehulling, and moist heat treatment (steaming or roasting) has produced precooked bean flours with reduced cooking times and improved nutritional profiles (59). These innovations not only save time and energy but also make beans more accessible and appealing to consumers worldwide.

### 3.3 Protein content

#### 3.3.1 Nutritional importance

Common beans serve as a vital plant-based protein source in diets, contributing to a reduced risk of chronic non-communicable diseases such as diabetes, cancer, obesity, and coronary heart disease (63). They are rich in various phytochemicals, including polyphenols, alkaloids, fiber, saponins, steroids, lectins, and terpenoids.

The proteins in common beans, primarily phaseolin and legumin, have shown the ability to inhibit HIV reverse transcriptase, thereby slowing viral progression. As a result, improving protein quality has become a key objective for common bean breeders, often taking precedence over yield improvements (1).

Common beans provide an economical protein source, particularly for low-income populations who may not afford more expensive animal protein options (63). They typically contain 15–25% protein on a dry weight basis, with water-soluble albumins and salt-soluble globulins constituting 10–30% and 45–70% of total proteins, respectively (65).

As a rich source of plant-based protein, common beans are increasingly important in meeting the growing dietary protein demands shifting toward plant-based sources (64). However, while they are an excellent protein source, common beans are low in sulfur amino acids, specifically methionine and cysteine, which affects their overall nutritional quality compared to animal proteins (65). Nonetheless, they contain high levels of lysine, an essential amino acid vital for collagen synthesis and maturation (67). This underscores the importance of a varied diet to ensure a complete amino acid profile.

Common beans are a valuable plant-based protein source that significantly contributes to human dietary protein intake. Their high protein content, along with other nutritional benefits such as dietary fiber, minerals, and bioactive compounds, makes them a crucial component of sustainable and healthy diets (68–70). As the demand for plant-based proteins continues to rise, common beans are poised to play an even more prominent role in global nutrition and food security (3).

#### 3.3.2 Variability in protein content

Common beans exhibit considerable variability in protein content, typically ranging from 20 to 25%, making these beans a significant source of plant-based protein (52, 122). This variability is modulated by complex genotypes-by-environment (GxE) interactions, influenced by both inherent genetic traits and external environmental conditions (71). Genetic factors are crucial in determining protein levels; for instance, the Higuera Azufrado variety is notable for its higher nitrogen, sulfur, and protein content (122). Additionally, the Pinto Saltillo cultivar demonstrates enhanced drought tolerance and shows upregulation of genes (e.g., *PvLEA3*) related to carbohydrate metabolism and cell wall dynamics under stress (72), indicating that certain varieties may possess genetic advantages for higher protein content and adaptability.

Environmental factors, particularly water availability, significantly impact protein content and overall bean quality. Drought conditions have been shown to alter gene expression in common beans, with the drought-tolerant Pinto Saltillo cultivar exhibiting 1,005 differentially expressed genes (72). Furthermore, genotype-environment interactions can significantly influence mineral content, as observed in Turkish common bean germplasm (73). However, such stress conditions may also alter amino acid profiles. Beyond protein, landrace varieties represent valuable genetic reservoirs, showing 15–30% higher levels of essential micronutrients like selenium and zinc compared to modern varieties, highlighting their significant potential for biofortification strategies (71).

While genetic factors primarily dictate the potential protein content of common bean varieties, environmental conditions play a critical role in expressing these traits. Breeding programs that focus on both genetic improvement and environmental adaptability are essential for developing high-protein, stress-tolerant bean varieties. Incorporating genomic selection and considering genotype-by-environment interactions in breeding strategies could accelerate the development of improved cultivars with enhanced protein content and resilience to various environmental stresses (74).

#### 3.3.3 Regional preferences and utilization

Common beans are a vital dietary protein source, particularly in protein-deficient regions, with protein content generally ranging from 20 to 25% (75, 76). In Brazil, the largest producer and consumer of common beans, they represent the primary source of dietary protein for many (75). Consumer preferences for specific bean varieties are heavily influenced by local adaptation and seed types. Growers and consumers often favor specific seeds, limiting breeding improvements to certain regions (76).

While protein content is a key consideration, other factors such as cooking time, water absorption, and the presence of antinutritional components also affect consumer choices (52). For example, although tepary beans (*Phaseolus acutifolius*) have similar protein levels to common beans, they may be less favored due to their challenging cooking characteristics, despite having lower antinutritional factors (52). Additionally, compounds that cause flatulence and antioxidants in common beans may further influence consumer preferences (77).

To enhance the nutritional value and acceptability of common beans in protein-deficient regions, breeding efforts should focus on improving protein quality, reducing antinutritional factors, and maintaining desirable cooking properties while considering local preferences for specific seed types and growth habits.

Table 1 below highlights global and regional disparities in daily protein intake and undernourishment, revealing Africa’s heavy reliance on plant protein (50.6 g vs. 15.7 g animal) and elevated undernourishment (19.9%), particularly in East/Middle Africa (46–59 g protein, ~29% undernourished). Common beans, rich in protein (~20–25%) and micronutrients, offer a strategic solution. Their drought resilience, affordability, and compatibility with African climates can address protein gaps, especially where animal protein is scarce. Cultivating biofortified varieties aligned with cultural preferences (e.g., yellow beans) and integrating beans into policies (subsidies, school meals) could enhance food security and reduce malnutrition sustainably.

**Table 1:** Comparison of daily protein intake (per capita) and undernourishment rates across global and African regions (110).

Region	Protein supply (g/cap/day)	Animal protein supply (g/cap/day)	Plant protein supply (g/cap/day)	Prevalence of undernourishment (%)
World	91.1	37.8	53.3	9.1
Africa	66.3	15.7	50.6	19.9
East Africa	59.4	11.9	47.5	29
Middle Africa	46.0	11.2	34.8	28.9
Southern Africa	79.7	38.4	41.3	9.4
Sub-Saharan Africa	60.7	13.0	47.7	22.7

#### 3.3.4 Challenges and opportunities

Common beans are a vital food source, especially in Africa and Latin America, providing essential nutrients such as protein, iron, and zinc. However, balancing protein content with yield and cooking quality poses significant challenges and opportunities. Drought stress, exacerbated by climate change, can severely impact bean yields and nutritional quality. Under drought conditions, yields may decrease by up to 56%, while protein and zinc levels can increase, but iron levels may decline (78). This trade-off emphasizes the need for climate-resilient varieties that can maintain both yield and nutritional quality.

Cooking time is another critical factor that affects bean utilization. Longer cooking times can limit consumption despite the nutritional benefits of beans (70). Breeding efforts have primarily focused on improving yields, often at the expense of quality and flavor traits (71). This presents an opportunity for collaboration among breeders, processors, and nutritionists to develop varieties that effectively balance protein content, yield, and cooking quality (70).

Research indicates that common beans can maintain their nutritional content in individual pods even under varying nutrient availability, showcasing resilience in reproductive tissues (79). This suggests potential for developing varieties that sustain protein levels under stress. Additionally, landraces have shown higher mineral content, particularly selenium and zinc, in comparison to modern varieties (71), representing valuable genetic resources for breeding programs focused on enhancing both yield and nutritional quality.

To tackle these challenges, various methods are being explored, including conventional breeding, transgenic approaches, and gene editing to improve iron and zinc accumulation in beans (80). Furthermore, the development of functionally enhanced common bean ingredients, such as protein-rich flours, offers opportunities to improve the nutritional profile of various food products without compromising sensory qualities (81).

### 3.4 Mineral content

#### 3.4.1 Key minerals in beans

Common beans are a significant source of essential minerals, particularly iron, zinc, and magnesium, contributing to dietary needs, especially in low-income regions. The mineral content in beans varies considerably, with iron concentrations ranging from 34 to 89 mg/kg and zinc levels between 21 and 54 mg/kg (82). Beans provide 23–30% of the daily recommended intake of iron, making them one of the best non-meat sources of this vital mineral (63).

However, the bioavailability of these minerals can be affected by antinutritional factors such as phytic acid and raffinose. Phytic acid chelates iron, reducing its bioavailability, while raffinose can cause digestive discomfort (1). Cooking methods also play a crucial role in mineral retention. Traditional and microwave cooking can decrease mineral content significantly, with reductions of 9.7–36.4% for calcium, 14.2–31% for iron, and 11.1–28.9% for zinc (83). Interestingly, industrially processed legumes often exhibit higher dialysability for calcium, iron, and zinc compared to those prepared traditionally or in microwaves (83).

While common beans are a rich source of essential minerals, their bioavailability can be hindered by antinutritional factors and cooking methods. To enhance mineral absorption, strategies such as biofortification (84), optimizing cooking techniques (85), and dietary diversification (86) are vital. Further research is necessary to understand the genetic mechanisms behind mineral accumulation in beans and to develop effective strategies for improving their nutritional quality.

#### 3.4.2 Regional deficiencies and bean consumption

Common beans play a critical role in addressing micronutrient deficiencies, particularly iron and zinc, in regions like Sub-Saharan Africa and South Asia. These areas face significant nutritional challenges, especially among young children and pregnant women (87, 88). Rich in essential minerals, common beans are a nutrient-dense food source that provides vital protein and micronutrients for millions across Latin America, the Caribbean, and Sub-Saharan Africa (46).

Biofortified varieties of common beans have shown promise in alleviating iron and zinc deficiencies in these regions (55). Research indicates that these enhanced beans can significantly improve micronutrient intake, addressing acute deficiencies and promoting better health among women and children (87).

Cooking methods and pre-treatment processes can influence the retention of iron and zinc in common beans. For instance, soaking beans before cooking can reduce overall cooking time and increase yield, but it may also impact mineral retention (47, 55). Despite these challenges, common beans remain an excellent source of iron and zinc. It is recommended to consume beans with their broth to maximize nutrient intake (47).

#### 3.4.3 Breeding for enhanced mineral content

Breeding efforts focused on biofortification have made significant strides in improving the mineral content of common beans, addressing global mineral deficiencies and enhancing human health. Research has demonstrated substantial variability in nutrient content among different bean accessions, providing a strong foundation for genetic improvement (89). Notably, quantitative trait loci (QTL) associated with iron and zinc concentrations have been identified, including a key overlapping QTL on linkage group b06, which may serve as a pleiotropic locus for mineral uptake or loading (90).

However, breeding for higher micronutrient content has been linked to increased levels of phytic acid, which can inhibit mineral absorption in the human body. To counteract this, low phytic acid (lpa) beans have been developed, exhibiting 90% lower phytic acid content than conventional beans (91). This creates an opportunity to combine lpa traits with biofortification efforts to enhance the nutritional benefits of these beans by reducing the phytic acid-to-iron and zinc ratio.

Genetic improvement initiatives have successfully produced bean varieties with increased mineral content, particularly for iron and zinc. The identification of QTLs and molecular markers related to mineral accumulation (89, 90, 92) provides valuable tools for marker-assisted selection in breeding programs. The integration of conventional breeding with modern biotechnological approaches offers a promising strategy to combat micronutrient deficiencies and improve global food security through biofortified common beans (93, 94).

### 3.5 Global and regional perspectives

#### 3.5.1 Regional differences in consumer preferences

Consumer preferences for common bean characteristics differ significantly across regions, influencing breeding efforts and market demand.

Seed coat color preferences are strong in various areas. For example, yellow beans are favored in some regions due to their association with faster cooking times (46). White and yellow varieties typically offer higher digestibility compared to red seed coat varieties (95). Conversely, dark-colored seeds, especially black beans, often contain higher levels of antioxidants and tannins (96). Factors such as seed coat color, hilum ring, and corona characteristics are important in determining consumer choice (25).

Cooking time is another critical trait affecting consumer preference. In regions where fuel costs are high, fast-cooking varieties are highly sought after (46). Genetic research has identified QTLs associated with shorter cooking times, with some varieties cooking up to 26 min faster than others (51). Additionally, beans grown in different environments can exhibit significant variations in cooking times; for instance, beans from Arusha can cook 15 min faster than those from Morogoro, Tanzania (51).

Protein and mineral content also influence regional preferences. Higher protein levels are generally desirable, and studies have found that QTLs for quicker cooking times often overlap with those for increased protein concentration in cooked seeds (51). Iron retention and bioavailability are particularly important in areas where beans are a primary nutritional source; fast-cooking yellow beans have demonstrated over 80% iron retention after boiling (46).

#### 3.5.2 Cultural and socioeconomic influences

Cultural and socioeconomic factors play a significant role in determining common bean preferences and consumption patterns across various regions. Common beans are a vital staple for food security and nutrition worldwide, especially in developing countries (78). Cultural norms, values, and social structures greatly influence the adoption and sustainability of agricultural technologies, including bean cultivation (97). For example, gender roles and labor divisions within communities affect participation in bean production, access to resources, and decision-making power, all of which impact productivity (97). In most of the bean farming communities, women are the primary cooks influencing demand for fast-cooking beans while men control market choices.

Although improved bean varieties may offer better yields or enhanced nutritional qualities, local preferences and customs can significantly influence their acceptance. Cooking time is a crucial factor affecting bean utilization; longer cooking times can limit consumption despite the crop’s nutritional benefits (70). Urbanization shifts preferences toward fast-cooking beans (12% decreased rural vs. 31% decreased urban cooking time in Tanzania); however, gender disparities persist—women prioritize cooking efficiency, while men control market choices (98). This highlights the need for breeding efforts to consider not only agronomic traits but also cultural practices and consumer preferences.

To fully leverage the potential of common beans in enhancing food security and nutrition, it is essential to foster collaboration among breeders, processors, and nutritionists (70). Market-driven approaches and gender-responsive participatory variety selection have proven effective in developing bean varieties that meet local demands in several African countries (98). These initiatives emphasize the importance of integrating cultural and socioeconomic factors into bean improvement programs to ensure widespread adoption and positive impact.

### 3.6 Challenges and future directions

#### 3.6.1 Challenges in meeting consumer demands

Balancing consumer preferences with yield and environmental adaptability presents significant challenges for common bean breeding. Historically, breeders have focused primarily on producers’ needs, often overlooking consumer requirements (99).

Phytate, a major antinutritional factor, acts as a potent chelator, binding minerals and proteins and forming insoluble complexes that reduce nutrient absorption in the intestines. This significantly lowers the bioavailability of essential minerals in the diet (1). This property poses health concerns for humans and non-ruminants like poultry and swine, which lack the digestive enzyme to break down phytate (100).

Common beans face numerous challenges in meeting consumer demands while maintaining yield and environmental adaptability. Breeding programs must address various biotic and abiotic stresses, including diseases, pests, drought, heat, cold, and nutrient deficiencies in soil, all of which significantly impact bean production (40). Climate change exacerbates these issues, potentially reducing productivity and threatening food security (101).

Efforts to enhance the nitrogen-fixing capacity of common beans have yielded mixed results. While some genotypes show improved nitrogen fixation at lower fertilizer levels, the correlation between nitrogen fixation and yield remains inconsistent (102). Additionally, climate change may lead to lower nutritional quality in beans, with reduced iron levels but increased protein and zinc content, along with elevated antinutritional compounds (78, 79).

To tackle these challenges, a multifaceted breeding approach is essential. Utilizing genetic resources from wild relatives and closely related species can introduce traits for stress tolerance and improved nutritional quality (103). Advanced genomic tools, such as high-density SNP marker arrays and next-generation sequencing, can expedite the identification of key loci associated with stress responses and consumer traits (101). Furthermore, considering local value chains and implementing demand-led breeding can help align consumer preferences with agronomic performance (40). By integrating these strategies, breeders can develop common bean varieties that satisfy consumer demands while ensuring yield stability and environmental adaptability.

#### 3.6.2 Role of modern breeding and biotechnology

Modern breeding and biotechnology innovations have significantly advanced the improvement of consumer traits in common beans, focusing on aspects such as seed coat darkening, cooking time, iron content, and protein levels.

Genomics and marker-assisted selection have been crucial in identifying and mapping quantitative trait loci (QTLs) associated with these traits (Table 2). For example, a genome-wide association study identified single-nucleotide polymorphisms (SNPs) on chromosomes Pv10 and Pv07 linked to seed coat color and hilum ring color, near genes involved in mature seed coat coloration (25). This information can guide targeted breeding efforts to meet consumer preferences for bean appearance.

**Table 2:** Summary of key genes/QTLs for target traits.

Trait	QTL/genomic region	Chromosome	Key findings	References
Protein content	Higuera Azufrado QTL		High nitrogen and sulfur content linked to elevated protein levels	Chávez-Mendoza et al. (122)
	PvB1-1	B1	Co-localizes with QTLs for seed weight and protein concentration	Cichy et al. (61)
Cooking time	CT-Pv03	Pv03	Reduces cooking time by 11–26 min; co-localizes with water absorption QTLs	Berry et al. (51)
	CT-Pv07	Pv07	Associated with faster cooking in Andean gene pool varieties. White-seeded genotypes	Diaz et al. (104)
	CT-Pv09	Pv09	Explains 15% phenotypic variance in cooking time	Berry et al. (51)
Iron content	Fe. Zn-b06	B06	Overlapping QTL for iron and zinc accumulation; pleiotropic locus	Blair et al. (90)
	Fe-Pv07	Pv07	SNP markers near anthocyanin biosynthesis enhance iron retention	Katuuramu et al. (105)
Iron bioavailability	FeBA-Pv11	Pv11	SNP explains 13.2% variation in iron bioavailability	Katuuramu et al. (105)
Seed coat darkening	*P*	Pv07	Master regulator of seed coat color; recessive *Psd* allele delays darkening	Islam et al. (10), Sadohara et al. (25)
	*J*	Pv01	Determines darkening potential (*jj* = non-darkening)	Elsadr et al. (21)
	*sd*	Pv07	Modulates darkening rate (*sdsd* = slow darkening)	Elsadr et al. (21)
	QTL-Pv10	Pv10	GWAS locus for seed coat lightness (L* value)	Sadohara et al. (25)

In relation to cooking time, research on 922 bean lines has pinpointed 10 QTLs associated with cooking time and water absorption capacity (104). The findings indicate that white-seeded beans cook the fastest, and genomic prediction models have shown promise in capturing genetic variation for cooking time, especially in the MAGIC population.

Regarding iron content and bioavailability, a study of 206 accessions from the Andean Diversity Panel identified significant SNP-trait associations that accounted for 6.3 to 13.2% of the phenotypic variation in seed protein, zinc, and calcium concentrations, as well as iron bioavailability (105). This research enhances the understanding of genetic architecture underlying these complex nutritional traits, facilitating genomics-assisted breeding efforts.

Some studies have revealed intriguing relationships between traits. For example, Diaz et al. (104) noted an inverse correlation between cooking time and water absorption capacity in Andean germplasm, with a specific QTL on Pv03 inversely controlling both traits. Additionally, Amongi et al. (106) observed minimal differences in mean performance across gene pools for most traits, except yield, where Mesoamerican beans outperformed their Andean counterparts.

Modern breeding and biotechnology innovations have greatly advanced our understanding of the genetic basis of consumer traits in common beans. Genomics and marker-assisted selection have facilitated the identification of key QTLs and SNPs associated with seed coat darkening, cooking time, iron content, and protein levels. These tools, combined with high-throughput genotyping platforms (107) and genomic selection approaches (25), are paving the way for more efficient breeding programs aimed at developing bean varieties with improved consumer traits. However, challenges remain, such as the complexity of certain traits and the necessity for further research to fully harness these technologies in bean improvement.

#### 3.6.3 Sustainability and climate change

Climate change poses significant threats to food security and crop productivity, particularly for staple crops like common beans. Research indicates that by 2050, many common bean-growing areas in southeastern Africa may become unsuitable for cultivation due to climate impacts (78). This situation underscores the urgent need to develop climate-resilient common bean varieties.

Strategies for creating climate-resilient beans include genetic modification and selective breeding for traits that enhance resilience to environmental stressors such as heat, drought, and salinity (108). These efforts aim to produce varieties that can better withstand adverse conditions (109). Additionally, integrating diverse cropping systems and introducing new crops can enhance the resilience of bean production (108).

While developing climate-resilient varieties is essential, it is also important to consider the potential impacts on nutritional quality. Research has shown that drought conditions induced by climate change may lead to decreased iron levels in common bean grains, while protein, zinc, and phytic acid levels may increase (78). This suggests that future bean servings could have diminished nutritional value, highlighting the need for a holistic approach that balances yield and nutritional quality in breeding programs.

Addressing climate change impacts on common bean production requires a multifaceted approach. This includes developing climate-resilient varieties through genetic methods, implementing biodiverse cropping systems, and ensuring that nutritional quality is factored into adaptation strategies. Tools like the Google Earth Engine geovisualization application (108) can help identify suitable areas for introducing these resilient varieties, facilitating sustainable and innovative agroecological solutions.

### 3.7 Future research

#### 3.7.1 Knowledge gaps

This review consolidates significant progress in understanding consumer-driven characteristics of common beans, highlighting how factors like seed coat darkening, cooking duration, and protein and mineral content influence global usage patterns. Our analysis indicates that these traits are not just agronomic issues but are crucial for market acceptance, nutritional security, and cultural significance, especially in regions with limited resources. Below, we provide context for key findings, discuss implications, and outline future priorities. Integration of Genetic, Biochemical, and Regional Insights We show that seed coat darkening, controlled by the J/sd/Psd genetic network and proanthocyanidin oxidation, directly affects marketability and nutrition. Slow-darkening (SD) varieties, such as pinto “1533-15,” offer a triple benefit: delayed discoloration, 30% faster cooking, and 2–7 × higher iron bioavailability (2, 8). This is consistent with Hamabwe et al. (7), who found reduced cooking times in Andean beans with stable seed coats. Importantly, regional preferences influence trait prioritization: East African consumers prefer yellow beans for quick cooking (6), while Latin American markets prioritize color stability in carioca beans (11). These differences necessitate customized breeding rather than one-size-fits-all solutions. Cooking Time: A Nexus of Nutrition and Socioeconomics The wide range in cooking times (19–271 min) highlights its role as a socioeconomic equalizer. Fast-cooking genotypes, like Manteca yellow, can cut fuel use by up to 50%—a crucial benefit in Sub-Saharan Africa, where 29% of the population is undernourished (110). Notably, we find an inverse relationship between cooking time and iron bioavailability (*r* = −0.76 (46)), challenging the belief that longer cooking improves nutrient access. This paradox underscores the need for genotype-specific processing guidelines. Recent innovations such as precooked flours (59) and UVC-assisted screening (33) provide scalable solutions, but regional adoption is uneven due to infrastructure limitations. Nutritional Trade-offs in a Changing Climate stressors create complex trade-offs: drought increases protein (15–25%) and zinc but reduces iron (78). Biofortification partially addresses this—low-phytic acid (lpa) beans enhance zinc/iron absorption by 90% (91), yet heat-induced hardening during storage can negate these benefits (49). Our synthesis supports Altaf et al. (73) focus on integrated biofortification, combining lpa traits with climate-resilient genes (e.g., drought-tolerant Pinto Saltillo). However, consumer acceptance challenges remain, particularly where antinutrients cause digestive issues (52). Future breeding must balance agronomic resilience with sensory quality, especially in regions where beans are a primary protein source (75). Limitations and Research Gaps While this review compiles global data, three gaps require urgent attention: (1) Bioavailability Mechanisms: The precise role of polyphenol-protein complexes in mineral chelation is still unclear. (2) Socioeconomic Drivers: There is a lack of quantitative models linking trait preferences to factors like gender, income, or urbanization. (3) Real-world Validation: Studies assessing nutrient retention in traditional recipes (e.g., fermented bean pastes) from field to plate are needed.

#### 3.7.2 Proposed directions

Mineral bioavailability remains a significant concern, particularly in developing countries, making the modulation of phytic acid content in seeds a key goal for genetic improvement in crops.

Future research on common beans should focus on several pivotal areas to address gaps in nutrient bioavailability and regional preferences:

1. Genetic mechanisms: Further investigation is needed into the genetic mechanisms that govern nutrient accumulation and bioavailability. While some studies have identified SNPs linked to zinc content (84) and other nutritional traits (105), a deeper understanding of the genetic architecture of these complex traits could enhance genomics-assisted breeding for biofortified bean varieties with improved nutrient profiles (80).
2. Gene editing: Utilizing CRISPR technology to target Psd and cooking-time QTLs (such as CT-Pv03 (51)) to speed up the development of new varieties.
3. Nutrient interactions: It is crucial to explore how different nutrients interact with antinutritional factors. Research indicates that polyphenols and phytates can inhibit iron bioavailability (111). More studies are needed to understand these interactions and how to mitigate their effects. Additionally, investigating the potential health benefits of certain antinutritional factors, such as their role in cancer prevention (68), could provide a more nuanced view of bean nutrition.
4. Regional preferences: Consumer preferences and cooking practices significantly impact nutrient retention and bioavailability. Research on fast-cooking bean varieties, such as the Manteca yellow bean (46), should be expanded to include other market classes and regions. This could help address the challenge of long cooking times, which can deter consumers and affect nutrient retention.
5. Innovative processing: Developing novel processing methods and food products using biofortified beans is another promising area for exploration. The creation of products like bean milk (112) and snacks made from lectin-free, low-phytic acid flour (113) demonstrates the potential for innovative bean-based foods. Further research in this area could promote increased bean consumption and improve nutrient delivery, especially in regions where beans are dietary staples.
6. Participatory breeding: Collaborating with farmers to design new varieties (for instance, Uganda’s gender-responsive initiatives (98)).
7. Policy integration: Ensuring bean improvement aligns with SDGs by providing subsidies for biofortified seeds and energy-efficient stoves.

While CRISPR editing of seed traits (e.g., *Psd* for slow-darkening seed coats) holds promise for rapid varietal development, scalable deployment in common bean remains constrained by transformation inefficiencies (114). Recent advances demonstrate that these bottlenecks can be mitigated through optimized gene-editing pipelines. For instance, transient transformation techniques like *hairy root editing* enable rapid validation of gene functions in common bean (115), and genotype-tailored delivery methods (e.g., *Agrobacterium* strain selection) significantly boost editing efficiency (116).

While genome editing remains a technical challenge in common beans compared to model crops, recent advancements in Brazil, the United States, and Africa, coupled with regional target product profiles (TPPs) and collaborative efforts like those of the Pan-Africa Bean Research Alliance (PABRA), provide strong evidence that genome editing is achievable on a regional scale.

Brazilian breeding programs have made significant strides in genomic technologies, including whole-genome resequencing of 40 elite bean lines, identifying over 420,509 high-quality SNPs for marker-assisted selection and potential genome editing targets. Preliminary studies on CRISPR/Cas9-mediated editing in common beans have targeted genes like Psd for slow-darkening seed coats, with successful edits reported in controlled settings (89).

The future research should adopt a multidisciplinary approach, combining genetics, nutrition, food science, and consumer preferences to develop biofortified bean varieties and products that are both nutritious and appealing to diverse populations worldwide. Initiatives like Pan-Africa Bean Research Alliance (PABRA) demonstrate scalable models: national programs co-develop varieties with farmers (e.g., gender-responsive selections in Uganda), while nutritionists integrate biofortification into school meals. Prioritizing ‘bean corridors’—linking high-yielding ecologies to protein-deficit zones—can attract investment amid legume competition. Demand-led varietal design and solution development are conducted using a multidisciplinary team approach which requires a broad range of competencies and actors with different roles and responsibilities to develop a new variety. Gender and other cross-cutting social dimensions should be well integrated into the generation, delivery and use of new technology (117).

The Pan-Africa Bean Research Alliance (PABRA) plays a crucial role in addressing the challenges and improving the productivity of common beans in Sub-Saharan Africa. PABRA collaborates with the International Center for Tropical Agriculture (CIAT) to conduct both strategic and applied research to address production risks like drought, which affects a significant portion of the bean production area in this region (118). The TPP, Product Development Team (PDT) and multi-stakeholder platform established within PABRA are pivotal for shaping the breeding programs and outputs. The TPP outlines key traits such as drought resistance, pest and disease resilience, and nutritional quality which are crucial for satisfying market demands and ensuring food security. This approach not only supports sustainable food systems but also strengthens food and nutrition security across the region (119).

The success of this model hinges on fostering public-private partnerships that can provide necessary support and resources to maintain supply chains efficiently. Nutrition-sensitive agriculture interventions were mainstreamed through the Pan-Africa Bean Research Alliance’s (PABRA) bean corridors approach between 2017 and 2023 (120). Also PABRA has developed a new “commodity corridors” approach, which aims to eliminate production bottlenecks, so that improved beans reach more consumers, and farmers can access better seeds (121).

## 4 Conclusion

Common beans serve as a prime example of how consumer preferences can connect agricultural and nutritional security. This review provides a novel system-oriented sysnthesis distinct from previous analysis by evaluating transnational evidence across Africa on scalable operational models—such as the co-development of regionally tailored, climate-smart varieties (e.g., SD beans, quick-cooking types, biofortified strains), their integration into nutrition interventions (e.g., school meals), and ‘bean corridor’ market linkages—we demonstrate how demand-driven approaches turn this ancient crop into a sustainable solution for 21st-century challenges. These models drive tangible improvements in nutrition security and livelihoods by centering the voices of farmers and consumers within interdisciplinary breeding innovations.
